# Innovation in hip preservation surgery

**DOI:** 10.1093/jhps/hnad025

**Published:** 2023-09-28

**Authors:** Richard E Field

**Affiliations:** Editor-in-Chief, Journal of Hip Preservation Surgery

Adopters of innovation have been described by Rogers [1] as belonging to one of the five groups: innovators, early adopters, early majority, late majority and laggards. In a perfect world, medical journals would provide a medium for innovators to report their discoveries, novel devices and new interventions. Independent, early-adopting, clinical researchers would rigorously evaluate these innovations and report their results. If supporting literature emerged, an early majority of clinicians would be persuaded to adopt the innovations. When unbiased researchers provided systematic reviews and meta-analyses confirming that the innovations provided superior clinical outcomes, the late majority would be able to advise their patients that the innovations were evidence-based, best practices. And, as time passed, the voice of the laggards would gradually fade. Better treatments would evolve, new skills would be acquired and medical expertise advanced.

At each step of the communication process, innovation needs to be presented in a manner that stimulates interest [2], and positive aspects need to be shown to outweigh drawbacks and limitations. It may take years to demonstrate superiority, resolve uncertainty and achieve a position where familiarity supersedes any awareness of novelty. In the early stages, adoption may be compromised for no better reason than overstated benefits shift attention to an innovation’s shortcomings and turn the community against what might have proved to be a useful development.

Maintaining interest in the incremental acquisition of evidence is not a process to which the human brain is well adapted. Attention to hazards and potential problems is programmed in our DNA and will always exceed our interest in familiar and safe activities [3]. While this trait may be advantageous for our survival, it means that we are more attentive to bad rather than good news [4]. Scrutiny of any media feed reveals a catalogue of international crises, conflicts and disasters, national problems and personal mishaps, falls from grace and failed interpersonal relationships. It is not our fault that a story about the disruption of a 60th wedding anniversary party is more newsworthy than six decades of partnership. There are a few subjects that may be exempt from the rule of ‘only bad things are interesting’. One is a sporting achievement and another is medical breakthroughs. Possibly because these reports offer hope that the frailties and vulnerability of the human condition can be overcome and the ‘long defeat’ [5, 6] of ageing may be prolonged.

Understanding these considerations is important when preparing a manuscript for publication. Whether it is to report an innovation, its scientific evaluation or scrutiny of accumulated evidence, the authors should focus on evidence rather than optimism. The authors must accept that their manuscript may be less interesting than a catalogue of clinical mishaps and failure. Few journals will ever publish papers that become known to the general public, and relatively few clinicians will prefer an evening reading their specialist journal over socializing with friends. Nevertheless, journals provide our repositories of shared knowledge and experience. In this regard, their value cannot be overstated and any effort required to acquaint ourselves with their content can only enhance our practice.

The development of extra-articular hip preservation surgery has paralleled the development of interventions for intra-articular interventions. Reports of hamstring tendon ruptures are not new [7], case series for open repair have been reported [8] and systematic reviews published [9]. However, endoscopic repair remains infrequent [10]. The potential benefits, limitations and hazards of endoscopic repair remain relatively unexplored so, Ranzoni’s paper [11] highlighting the proximity of endoscopic portal tracts to the posterior femoral cutaneous nerve provides a valuable contribution to this potential innovation in the treatment of hamstring injuries.

This issue includes two systematic reviews. The paper from Banks [12] identifies that, over more than two decades, the hip preservation community has provided relatively few robust studies on post-operative pain management for hip preservation procedures. The authors do confirm that peripheral nerve blocks are associated with a significant reduction in pain scores when compared with general anaesthesia but also identify that their review included a spectrum of hip preservation operations. They suggest that more work is needed to identify optimal strategies for the different interventions undertaken by hip preservation surgeons before we can advise patients on best practices.

The paper from Curley [13] investigates the innovation of computer-assisted peri-acetabular osteotomy (PAO). While the authors recognize that the application of computer-assisted technology to PAO surgery is in its infancy and considerable heterogenicity exists between the techniques under evaluation, they do confirm improvements in patient-reported outcomes and a reduction in intraoperative exposure to ionizing radiation. While our successors may be amazed that freehand PAO surgery was once the norm, for now, the adoption of computer-assisted technology remains the province of innovators and, perhaps, a few early adopters.

I hope that you enjoy all the papers in Issue 10.2 and that you will be inspired to innovate, evaluate and report your work in the *Journal of Hip Preservation Surgery*.
